# Willingness to Accept HIV Pre-Exposure Prophylaxis among Chinese Men Who Have Sex with Men

**DOI:** 10.1371/journal.pone.0032329

**Published:** 2012-03-30

**Authors:** Feng Zhou, Lei Gao, Shuming Li, Dongliang Li, Lifen Zhang, Wensheng Fan, Xueying Yang, Mingrun Yu, Dong Xiao, Li Yan, Zheng Zhang, Wei Shi, Fengji Luo, Yuhua Ruan, Qi Jin

**Affiliations:** 1 Institute of Pathogen Biology, Chinese Academy of Medical Sciences & Peking Union Medical College, Beijing, China; 2 Beijing Chaoyang Center for Disease Control and Prevention, Beijing, China; 3 Department of Epidemiology and Statistics, Institute of Basic Medical Sciences, Chinese Academic of Medical Sciences and School of Basic Medicine, Peking Union Medical College, Beijing, China; 4 Department of Public Health, College of Health and Human Service, Western Kentucky University, Bowling Green, Kentucky, United States of America; 5 State Key Laboratory for Infectious Disease Prevention and Control, and National Center for AIDS/STD Control and Prevention (NCAIDS), Chinese Center for Disease Control and Prevention, Beijing, China; 6 Chaoyang Chinese AIDS Volunteer Group, Beijing, China; 7 Beijing Jingcheng Skin Diseases Hospital, Beijing, China; 8 Beijing Chaoyang Health Bureau, Beijing, China; Lady Davis Institute for Medical Research, Canada

## Abstract

**Objective:**

We investigated the awareness and acceptability of pre-exposure prophylaxis (PrEP) among men who have sex with men (MSM) and potential predicting factors.

**Methods:**

This study was conducted among MSM in Beijing, China. Study participants, randomly selected from an MSM cohort, completed a structured questionnaire, and provided their blood samples to test for HIV infection and syphilis. Univariate logistic regression analyses were performed to evaluate the factors associated with willingness to accept (WTA) PrEP. Factors independently associated with willingness to accept were identified by entering variables into stepwise logistic regression analysis.

**Results:**

A total of 152 MSM completed the survey; 11.2% had ever heard of PrEP and 67.8% were willing to accept it. Univariate analysis showed that age, years of education, consistent condom use in the past 6 months, heterosexual behavior in the past 6 months, having ever heard of PrEP and the side effects of antiretroviral drugs, and worry about antiretroviral drugs cost were significantly associated with willingness to accept PrEP. In the multivariate logistic regression model, only consistent condom use in the past 6 months (odds ratio [OR]: 0.31; 95% confidence interval [CI]: 0.13–0.70) and having ever heard of the side effects of antiretroviral drugs (OR: 0.30; 95% CI: 0.14–0.67) were independently associated with willingness to accept PrEP.

**Conclusions:**

The awareness of PrEP in the MSM population was low. Sexual behavioral characteristics and knowledge about ART drugs may have effects on willingness to accept PrEP. Comprehensive prevention strategies should be recommended in the MSM community.

## Introduction

In pre-exposure prophylaxis (PrEP), antiretroviral (ARV) drugs are given to HIV-negative people to decrease their chance of becoming infected. Several studies conducted among men who have sex with men (MSM) have shown that PrEP awareness was very low, and few participants reported having the experience of PrEP use, even in some countries where it is available [1]–[3]. Although strategies including abstinence, being faithful, and condom use (ABC) have been proved to be effective for prevention of HIV transmission, the virus still prevails among MSM. It was estimated that 2.6 million individuals were newly infected in 2009 worldwide, which 19% fewer than the 3.1 million in 1999 [4]. China had about 740,000 people living with HIV and 105,000 with AIDS by the end of 2009 [5]. Homosexual intercourse has become a major mode of HIV transmission since 2009, and the prevalence of HIV in MSM has increased significantly from 2.5% in 2006 to 8.6% in 2009 [5]. A sociological study has estimated that there are 1.8–2.4 million homosexual or bisexual men in mainland China [6]. In China, high-risk behavior, such as multiple partners and unprotected sex, have been reported to be common in this group [7]–[14]. Also, recent studies have reported rapid transmission of HIV in this specific population from various geographic areas in China, despite the efforts made by the national and local governments and non-governmental organizations in the past few years [15]–[18]. New effective approaches are urgently needed for this population.

In recent decades, researchers have made great efforts to explore alternative biomedical interventions, such as male circumcision (MC), HIV PrEP and post-exposure prophylaxis (PEP), HIV vaccines, and microbicides. Among these potential strategies, PrEP is considered to be one of the most promising strategies in MSM. Several animal and human studies have suggested that ARV drugs might reduce the risk of HIV infection either by PrEP or by non-occupational PEP [10], [15], [19], [20]. A 12-month PrEP clinical trial of daily oral tenofovir disoproxil fumarate (TDF) for HIV prevention was performed among 400 HIV-negative Ghanaian women, and achieved good acceptability and >82% adherence [21]. In November 2010, the US National Institutes of Health (NIH) announced the results of the iPrEx trial of PrEP conducted among 2499 HIV-seronegative MSM in six countries, which showed that daily oral Truvada, a combination of emtricitabine (FTC) and TDF, reduced risk of HIV incidence by 44%, with a median 1.2 years follow-up, compared with the placebo group, and >75% adherence was reached [22]. These findings represent a major advance in HIV prevention research, providing the first evidence that PrEP, when combined with other prevention strategies, can reduce HIV risk among MSM. A further study is ongoing in HIV Prevention Trials Network (HPTN) 067 to evaluate the feasibility of intermittent dosing of PrEP. Recent results from Partners PrEP and CDC TDF2 have shown that PrEP with daily oral TDF/FTC or TDF was effective at reducing HIV risk in heterosexual men and women [23], [24]. However, the Fem-Prep program on Truvada, a closed clinical trial implemented by Family Health International (FHI) in partnership with research centers in Africa, does not support the theory of PrEP having an effect on HIV prevention [25]. Therefore, some factors that might influence the efficacy of PrEP, including adherence, sexual behavior, or other factors still need to be determined.

The awareness and acceptability of new strategies are very important when they are recommended for use. Therefore, the objective of our study was to investigate the awareness and acceptability of PrEP among MSM and potential impact factors, which will provide suggestions and guidelines for future clinical trials in China.

## Results

### Demographic characteristics

A total of 159 participants were enrolled in the study. Seven were deleted because of not having sex with men in the past 6 months, by self-reports. Finally, 152 were used for the analyses. Age of the participants ranged from 18 to 61 years, with a mean age of 29.7±8.6 years. One hundred and three (67.8%) subjects expressed willingness to definitely or probably take PrEP if it were available in China in the future. In the univariate logistic regression model, age and years of education were found to be associated with WTA (Table 1).

**Table 1:** Associations between demographical characteristics and willingness to accept PrEP.

Factors	WTAEvent/Total (%)	OR (95% CI)	p value
Age			
<30 years	66/96 (68.8)	1.00	
≥30 years	47/56 (83.9)	2.37 (1.03, 5.46)	0.04
Marital status			
Single/divorced/widowed	76/104 (73.1)	1.00	
Married/cohabitated	37/48 (77.1)	1.24 (0.56, 2.76)	0.60
Registered Beijing residence			
No	94/122 (77.1)	1.00	
Yes	19/30 (63.3)	0.52 (0.22, 1.21)	0.13
Han Ethnic			
No	8/10 (80.0)	1.00	
Yes	105/142 (73.9)	0.71 (0.14, 3.49)	0.67
Years of education			
<12	64/80 (80.0)	1.00	
≥12	49/72 (68.1)	0.53 (0.26, 1.12)	0.09
Monthly income (RMB)			
≤2000	54/70 (77.1)	1.00	
>2000	59/82 (72.0)	0.76 (0.36, 1.59)	0.47
Good HIV/AIDS knowledge			
No	55/71 (77.5)	1.00	
Yes	58/81 (71.6)	0.73 (0.35, 1.53)	0.41

Abbreviation: CI, confidential interval; OR, odds ratio; PrEP, pre-exposure prophylaxis; WTA, willingness to accept.

### Behavioral characteristics and laboratory test results

Homosexual men accounted for 84.9%, and bisexual men for 15.1% of the selected population, and 53.3% of them were using condoms consistently. The median number of their homosexual partners was three before baseline. In the past 6 months, insertive or predominantly insertive anal sexual intercourse was reported by 101 participants (66.4%) and receptive or predominantly receptive anal sexual intercourse was reported by 51 participants (33.6%); eight participants (5.3%) had received money for sex from male partners, and five (3.3%) had provided money for sex to male partners; 49.3% reported having bought condom lubricant. All of the participants were tested for HIV infection or syphilis and none of them were found to be infected. In the univariate regression model, inconsistent condom use in homosexual behavior in the past 6 months and heterosexual behavior in the past 6 months were shown to be associated significantly with WTA (Table 2).

**Table 2:** Associations between sexual behaviors and willingness to accept PrEP.

Factors	WTAEvent/Total (%)	OR (95% CI)	p value
Sexual orientation			
Bisexual or uncertain	42/57 (73.7)	1.00	
Homosexual	71/95 (74.7)	1.06 (0.50, 2.24)	0.89
Total number of MSM friends			
<10	48/68 (70.6)	1.00	
≥10	65/84 (77.4)	1.43 (0.69, 2.96)	0.34
Age at the first insertive sexual intercourse			
<20 years	36/50 (72.0)	1.00	
≥20 years	77/102 (75.5)	1.20 (0.56, 2.57)	0.64
Gender of the first sexual partner			
Male	69/97 (71.1)	1.00	
Female	44/55 (80.0)	1.62 (0.73, 3.59)	0.23
Styles of homosexual behavior in the past 6 months			
Receptive or predominantly receptive	38/51 (74.5)	1.00	
Insertive or predominantly insertive	75/101 (74.3)	0.99 (0.46, 2.14)	0.97
Seeking sexual partners via internet			
No	49/64 (76.6)	1.00	
Yes	64/88 (72.7)	0.82 (0.39, 1.72)	0.59
Only one male sexual partner in the past 6 months			
No	94/122 (77.1)	1.00	
Yes	19/30 (63.3)	0.52 (0.22, 1.21)	0.13
Diagnosed as STDs in the past 6 months			
No	97/134 (72.4)	1.00	
Yes	16/18 (88.9)	3.06 (0.67, 13.92)	0.15
Consistent condom use in homosexual behaviors in the past 6 months			
No	60/71 (84.5)	1.00	
Yes	53/81 (65.4)	0.35 (0.16, 0.76)	0.01
Having bought condom lubricant in the past 6 months			
No	58/77 (75.3)	1.00	
Yes	55/75 (73.3)	0.90 (0.44, 1.87)	0.78
Heterosexual behaviors in the past 6 months			
No	92/129 (71.3)	1.00	
Yes	21/23 (91.3)	4.22 (0.94, 18.92)	0.06
Having received money for sex from male partners			
No	109/147 (74.2)	1.00	
Yes	4/5 (80.0)	1.39 (0.15, 12.86)	0.77
Having given money for sex to male partners			
No	105/144 (72.9)	1.00	
Yes	8/8 (100.0)	–	–

Abbreviation: CI, confidential interval; OR, odds ratio; PrEP, pre-exposure prophylaxis; STD, sexually transmitted diseases; WTA, willingness to accept.

### Potential benefits/risks from PrEP

Seventeen participants (11.2%) had ever heard of PrEP before our study; 69.1% reported having ever heard that ARV drugs can help control AIDS development; and 32.9% reported having ever heard of side effects of ARV drugs. With regard to the potential risks of PrEP, 63.8% expressed being worried about not working due to the side effects from PrEP; 44.1% expressed worry that PrEP has no prevention efficacy; 44.7% expressed worry about diet and sleep being disrupted by PrEP; 21.7% expressed worry about drug resistance from PrEP; 20.1% expressed worry about being treated as an AIDS patient by people; 14.5% expressed worry about being refused sex by male partners after using ARV drugs; and 26.3% expressed worry about not being able to afford ARV drugs. Univariate logistic regression found that having ever heard of PrEP and the side effects of ARV drugs, and being worried about not being able to afford ARV drugs were significantly associated with WTA (Table 3).

**Table 3:** Associations between perceived PrEP benefits/risks and willingness to accept PrEP.

Factors	WTAEvent/Total (%)	OR (95% CI)	p value
Having ever heard of PrEP*			
No	97/135 (71.9)	1.00	
Yes	16/17 (94.1)	6.27 (0.80, 48.92)	0.08
Having ever heard that ARV drugs can help control AIDS development			
No	33/47 (70.2)	1.00	
Yes	80/105 (76.2)	1.36 (0.63, 2.93)	0.44
Having ever heard of side effects on ARV drugs*			
No	83/102 (81.4)	1.00	
Yes	30/50 (60.0)	0.34 (0.16, 0.73)	0.01
Having worry not to work due to the side effects from PrEP			
No	38/55 (69.1)	1.00	
Yes	75/97 (77.3)	1.53 (0.73, 3.21)	0.27
Having worry that PrEP have no prevention efficacy			
No	63/85 (74.1)	1.00	
Yes	50/67 (74.6)	1.03 (0.49, 2.14)	0.94
Having worry diet and sleep to be interrupted for PrEP			
No	61/84 (72.6)	1.00	
Yes	52/68 (76.5)	1.23 (0.59, 2.56)	0.59
Having worry drug resistance from PrEP			
No	87/119 (73.1)	1.00	
Yes	26/33 (78.8)	1.37 (0.54, 3.46)	0.51
Having worry to be treated as a AIDS patient by people			
No	90/122 (73.8)	1.00	
Yes	23/30 (76.7)	1.17 (0.46, 2.98)	0.75
Having worry to be refused having sex by male sexual partners after using ARV drugs*			
No	96/130 (73.9)	1.00	
Yes	17/22 (77.3)	1.20 (0.41, 3.51)	0.73
Having worry not to afford the ARV drugs			
No	79/112 (70.5)	1.00	
Yes	34/40 (85.0)	2.37 (0.91, 6.17)	0.08

Abbreviation: ARV, antiretroviral; AIDS, Acquired Immune Deficiency Syndrome; CI, confidential interval; OR, odds ratio; PrEP, pre-exposure prophylaxis; WTA, willingness to accept.

### Results from multivariate analyses

All of the variables associated with WTA in univariate analyses were included in a multivariate logistic regression model and were applied for variable selection stepwise to determine a final model. In the multivariate logistic regression model, those who did not have consistent condom use in homosexual behavior in the past 6 months (OR: 0.31; 95% CI: 0.13–0.70), and had never heard of the side effects of ARV drugs (OR: 0.30; 95% CI: 0.14–0.67) were willing to accept PrEP (Table 4).

**Table 4:** The associations of willingness to accept PrEP with potential factors in multivariate logistic regression model.

Factors	Multivariate OR (95% CI)	p value
Consistent condom use in homosexual behaviors in the past six months		
Yes vs. No	0.31 (0.13, 0.70)	<0.01
Having ever heard of side effect of ARV		
Yes vs. No	0.30 (0.14, 0.67)	<0.01

Abbreviation: ARV, antiretroviral; CI, confidential interval; OR, odds ratio; PrEP, pre-exposure prophylaxis.

## Discussion

To our knowledge, this is the first study to assess the awareness and acceptability of PrEP among MSM in China, and potential factors associated with the willingness to accept PrEP were also evaluated. Awareness of PrEP in MSM was rather low, only accounting for 11.2%. However, 67.8% reported that they were definitely or probably willing to accept PrEP if available in China. Those who did not practice consistent condom use and had never heard of the side effects of ARV drugs were more willing to accept PrEP.

Antiretroviral prophylaxis has been shown to be effective in preventing HIV transmission among MSM, pregnant women, and HIV-discordant couples in recent clinical trials [22], [28]–[30]. However, some issues still need further research, such as long-term effects of using PrEP with regard to drug resistance, the side effects of PrEP drugs, and adherence. These issues may affect the willingness to take PrEP. In the past several years, several studies have been conducted among MSM to investigate their attitudes towards HIV biomedical prevention technologies in developed countries [1]–[3]. However, such studies have not been conducted in China. The awareness of PrEP in our study was lower (11.2%) compared to previous studies from the United States (16–23.2%) [2], [31], [32]. The most plausible explanation might be knowledge limitation and source limitation of ARV drugs in China. Although PrEP was unfamiliar in China, the potential for PrEP generalization still seemed highly feasible, and a 67.8% WTA rate could be considered as a moderate level.

Education levels and knowledge about PrEP and ARV drugs may also have affected the WTA of this strategy. Studies have shown that MSM who have less education and knowledge about PrEP and ARV drugs are likely to start using PrEP for HIV prevention [2], [31], which is consistent with our study. A qualitative study that has investigated initial commitment to PrEP and MC among Indian truck drivers has suggested that cultural beliefs towards medication and physicians, and cost of HIV preventive interventions may affect WTA [33]. Although there was no significant effect of cultural factors on WTA in our study, the feasibility of PrEP in different cultural, ethical, legal and political contexts is still one of the key action points in planning for PrEP. Preventing HIV is complex and involves many social issues [34]. Therefore further studies should be conducted in the process of preparing for PrEP.

PrEP should never be seen as the first line of defense against HIV, because it has only been shown to be effective in clinical trials when provided in combination with regular HIV testing, condoms, and other proven prevention methods. One study that showed that more than 35% of those who expressed willingness to accept PrEP use for HIV prevention reported that they would be likely to decrease condom use during PrEP use [32]. Our results indicate that participants who do not insist on using condoms in homosexual behavior showed higher willingness to accept PrEP. These results showed the competitive selection between PrEP and condom use that might occur because people are more prone to use a simple and effective way to solve a problem. Therefore, combination of consistent condom use and PrEP, even including other confirmed efficient strategies, should not be ignored when PrEP is introduced.

Several limitations of this study should be kept in mind. First, potential bias about sensitive questions could not be excluded definitely because our questionnaires were interviewer-administered. Second, non-probability sampling methods might have induced potential selection bias and therefore limited the generalization of the study findings to the whole MSM population in China. Third, the willingness to change was also an important issue, which could not be ignored. If other new biomedical prevention interventions were also available in China, study participants would balance the benefits and risks from each intervention.

## Methods

### Study design and participants

The study aimed to explore the acceptability of new biomedical prevention strategies, including MC, HIV PrEP, HIV vaccines, and microbicides among the MSM population in Beijing. Recruitment was based on a seroepidemiological cohort study on HIV incidence among HIV-negative MSM in Beijing. Nine hundred and twenty-six potential participants were enrolled and 808 were eligible and agreed to participate, between August 2009 and January 2010. Inclusion criteria of the cohort study were age ≥18 years, male sex, having had sex with other men in the past 6 months, HIV-negative or unknown status by self-reports, being willing to participate and provide written informed consent. Eligible subjects returned to the clinic every 3 months. Structured interviewer-administered questionnaires and blood samples were collected at baseline and at each follow-up. The cohort study participants were recruited using convenience sampling through three approaches. First, study participants were recruited through website advertisements in the charge of a non-governmental AIDS volunteer group (www.hivolunt.net). Second, peer educators were hired and trained, who were responsible for distributing cards including information about our study at MSM-frequented venues, such as MSM clubs, bars, parks and bathhouses. Third, subjects were encouraged to introduce MSM around them to participate in the study [26]. Participants were randomly selected from this cohort at the first follow-up visit between November 2009 and April 2010. Participants were separated into four units according to the ascending order of study identification numbers. Each unit had 25 participants. Units were subsequently distributed into four groups, which were asked to complete questionnaires about the willingness to accept MC, microbicides, HIV vaccine, or PrEP. Only the willingness to accept PrEP is focused upon in this paper. An introductory statement about our program was written in Chinese on the website or printed materials. To keep participant information confidential, actual names were not required, so nicknames were permitted when signing the informed consent forms. After obtaining written informed consent, trained health workers provided a brief introduction about PrEP for one MSM or a group. PrEP explanation was translated into English as follows.

“PrEP means pre-exposure prophylaxis, which means to take some medicine for preventing a disease before risk exposure. ARV drugs have been suggested effective and safe to treat AIDS and regularly applied in treatment of HIV/AIDS patients in the past several years. Currently, foreign and domestic scientific researchers are making efforts to generalize some ARV drugs into HIV-negative risk population before HIV exposure. In this survey, you are invited to answer some questions about your awareness and willingness to accept future PrEP intervention.”

After the explanation, a structured questionnaire was administered by a trained interviewer for each participant in a private room. ARV drugs used in PrEP were described after assessing the awareness of PrEP, but before asking the questions about the willingness to accept PrEP, because most participants might have never heard of PrEP, TDF and FTC in China. The details of PrEP were translated into English as follows.

“Several clinical trials using TDF and/or FTC for PrEP are ongoing or have been completed in some countries. There are two suggested regimens and doses so far, which were recommended for clinical trails of PrEP. One is one-drug PrEP (TDF), whose suggested dose is tenofovir disoproxil fumarate (TDF) 300 mg once a day; the other is two-drug PrEP (TDF/FTC), and suggested dose is TDF/FTC 300 mg/200 mg once a day. Both drugs have possible side effects, such as nausea, vomiting, headache etc, similar to other ARV drugs, but it would be improved after a few weeks of PrEP use. Unlike ARV drug treatment for AIDS patients, however, treatment withdrawal and resistance prevention should be considered simultaneously. No definite strategies are available so far for PrEP clinical research. Although there are still some challenges in PrEP research, it is still one promising strategy in HIV prevention. In this study, we want to know whether you want to accept it and have worries about it, if PrEP is proved to be safe and effective.”

Participants were asked their willingness to accept PrEP. Some potential predictors were also investigated, which might influence PrEP acceptability and desirable information acquisition approaches. Blood samples were collected for HIV and syphilis testing after each questionnaire interview. Confidential HIV pre-test counseling was conducted and post-test counseling was also provided when participants returned to clinics to hear the results of their HIV test. The study protocol and informed consent were approved by the institutional review board of Beijing Chaoyang District Center for Disease Control and Prevention.

### Data collection

Each participant was assigned an exclusive identification number that was used to link the questionnaire and specimens. Face-to-face interviews were conducted in a private room. The content of the structured questionnaire included sociodemographic information (i.e. age, marital status, registered Beijing residence, ethnics, years of education, monthly income), HIV/AIDS knowledge (i.e. five indicators of UN General Assembly Special Session on HIV/AIDS, UNGASS, including HIV can be avoided by having sex with only one faithful, uninfected partner; HIV can be avoided by using condoms; a healthy looking person can have HIV; a person cannot acquire HIV from mosquito bites; a person cannot acquire HIV by sharing a meal with someone who is infected [27]), sexual behavior (i.e. sexual orientation, total number of MSM friends, age at first insertive sexual intercourse, sex of the first partner, styles of homosexual behavior in the past 6 months, seeking sexual partners via the internet, only one male sexual partner in the past 6 months, diagnosed with sexually transmitted disease in the past 6 months, consistent condom use in homosexual behavior in the past 6 months, having bought condom lubricant in the past 6 months, heterosexual behavior in the past 6 months, having received money for sex from male partners, having given money for sex to male partners). Participants were also asked about awareness and use of ARV drugs for PrEP, the likelihood of using PrEP in the future, and their worries about PrEP. Willingness to accept PrEP was assessed on four scales, which were translated into English as follows.

- “I am definitely willing to take PrEP.” [Definitely yes];
- “I am probably willing to take PrEP.” [Probably yes];
- “I am probably not willing to take PrEP.” [Probably no];
- “I am definitely not willing to take PrEP.” [Definitely no]

### Laboratory tests

HIV infection status was determined by an enzyme immunoassay (EIA) (Shanghai Kehua Bio-Engineering Co. Ltd., China) and an HIV-1/2 Western Blot confirmation (HIV Blot 2.2 WBTM, Genelabs Diagnostics, Singapore). Syphilis infection was determined using an EIA (Beijing Kinghawk Pharmaceutical Co. Ltd., China) and confirmed with a Passive Particle Agglutination Test for Detection of Antibodies to *Treponema pallidum* (TPPATM, FUJIREBIO, Japan).

### Data analysis

Data were double-entered and made consistent using EpiData version 3.1 (EpiData Association, Odense, Denmark). Those who were definitely or probably willing to accept PrEP were combined as willingness to accept (WTA), and those who were probably not or definitely not willing to accept PrEP were combined as non-willingness to accept (non-WTA). Those who gave all five correct answers to UNGASS questions were considered to be aware of the HIV/AIDS-related knowledge. Univariate logistic analysis was performed to evaluate the associations of WTA with the characteristics of demographics, sexual risk behavior, and perceived PrEP benefit/risk. Factors associated with WTA were identified by entering variables with P values<0.1 into stepwise logistic regression analysis. The statistics software SAS version 9.2 (SAS Institute Inc., Cary, NC, USA), was used for the analyses.
