# Correction: Willingness to Accept HIV Pre-Exposure Prophylaxis among Chinese Men Who Have Sex with Men

**DOI:** 10.1371/annotation/8d587ce6-3695-4028-8f2d-3a3c10d489b6

**Published:** 2012-10-05

**Authors:** Feng Zhou, Lei Gao, Shuming Li, Dongliang Li, Lifen Zhang, Wensheng Fan, Xueying Yang, Mingrun Yu, Dong Xiao, Li Yan, Zheng Zhang, Wei Shi, Fengji Luo, Yuhua Ruan, Qi Jin

The funding project numbers were omitted from the published Funding statement. The correct Funding is:

This work was supported by the grants from the Ministry of Science and Technology of China (2009ZX10004-903, 2008ZX10001-016, 2008ZX10001-004 and 2009DFB30420). The funders had no role in study design, data collection and analysis, desicion to publish, or preparation of the manuscript.

